# Diversity and prevalence of gastrointestinal parasites of Black Bengal goats in Natore, Bangladesh

**DOI:** 10.5455/javar.2023.j655

**Published:** 2023-03-31

**Authors:** Mita Chakrabortty, Nusrat Nowrin Shohana, Nurjahan Begum, Anita Rani Dey, Sharmin Aqter Rony, Shirin Akter, Mohammad Zahangir Alam

**Affiliations:** Department of Parasitology, Faculty of Veterinary Science, Bangladesh Agricultural University, Mymensingh, Bangladesh

**Keywords:** Black Bengal goats, gastrointestinal, parasite, Natore

## Abstract

**Objectives:**

The objective of this work was to estimate the diversity of gastrointestinal (GI) parasite species, their prevalence, and risk factors in Black Bengal goats (BBGs) of Natore, Bangladesh.

**Materials and Methods:**

Fecal samples from randomly selected 260 BBGs were processed through Stoll’s ova counting method, floatation, and simple sedimentation method. Microscopy-based identification of parasitic eggs, cysts, or oocysts was made. A semi-structured questionnaire-based data on host and management practices were collected from the owner. Data analysis was done using Statistical Package for Social Sciences.

**Results:**

The overall prevalence of GI parasites in BBGs was 65.4%, with an individual prevalence of 8.5% for *Fasciola gigantica, *21.5% for *Paramphistomum *spp., 20% for *Haemonchus* spp., 34.2% for *Strongyloides *spp., 8.5% for *Trichuris *spp., and 9.2% for *Eimeria *spp. No significant effect of host age, gender, body condition, animal rearing system, or housing floor type was observed on parasitism. Animals of young age, female, poorly body-conditioned, living in a free-range system, and housed on a muddy floor had a relatively higher susceptibility to infection. Deworming had a significant impact on reducing the frequency of caprine GI parasitism.

**Conclusions:**

Despite the significant effect of anthelmintic, the elevated prevalence of GI parasites in BBGs suggests a critical need for developing effective strategies to prevent caprine parasitoses.

## Introduction

Livestock occupies 1.90% of the gross domestic product with a growth rate of 3.10% and a goat population size of 267.74 million in Bangladesh [1]. The number of registered goats was 56,000 in Bangladesh, which offers employment for 281,000 people [2]. Small ruminant rearing, like goat raising, is a popular traditional business in Bangladesh and contributes greatly to the people living below the poverty line. Of the total goat population, 90% are Black Bengal goats (BBGs) [2]. The BBG is the most famous goat breed in Bangladesh as they are easily adaptable, highly prolific, and productive [3,4]. They are capable of resisting tropical diseases and producing quality meat, milk, and skin [5,6]. Faulty rearing strategies are responsible for excess treatment costs and loss of production, ultimately leading to reduced profitability for farmers [7]. Parasitism is an important limiting factor in Bangladesh [8] because of the favorable geo-climatic conditions, including the water-logged and low-lying areas, which increase the fecundity of various parasites. [9,10]. Natore district is a typical example of riverine Bangladesh because of its extensive network of natural water resources, including Padma, Atrai, Baral, Nagar, Baranai, Gurh, and exclusively *Chalan Beel* [11]. Being a wetland and floodable area, Natore could be considered a geotropically vulnerable zone for farm animal parasitosis.

Among various parasites, goats are chiefly the victims of gastrointestinal (GI) helminths. The principal GI parasites responsible for loss of productivity in BBGs include *Haemonchus, Trichostrongylus*,* Oesophagostomum, Strongyloides*,* Trichuris, *hookworm, *Moniezia, Paramphistomum, Fasciola, *and *Schistosoma *[9,12,13]. *Eimeria* spp. prevail worldwide and cause either a clinical or subclinical form of coccidiosis in small ruminants [14,15]. Young animals are mostly affected in stressed conditions, and pathogenic significance includes enteric disease and, ultimately, death [14,15]. *Taenia* *hydatigena, *a cestode parasite, spends a part of its life cycle in goats as an intermediate host, which has both economical and clinical importance [16]. A high prevalence of GI helminths has been recorded in multiple areas of Bangladesh. Nath et al. [17], Hassan et al. [18], Rabbi et al. [19], Akther et al. [20], Bhowmik et al. [21], and Hossain et al. [12] reported a prevalence of 94.67% in Chottogram, 63.41% in Chottogram, 76.5% in 4 districts (Mymensingh, Tangail, Netrakona, and Jaypurhat), 9.77% in Dinajpur, 61.82% in Sandwip, Chottogram, 51.1% in Mymensingh in goats of respective areas. Dey et al. [9] also found a 62.1% prevalence of GI nematodes in different topographic regions of Bangladesh. All these reports indicate that goats are highly vulnerable to parasitism in this country.

Changes in parasite levels bring changes in animal health products, ultimately affecting the global livestock system [22]. From the context of the voluminous literature on parasite prevalence, it can be assumed that 70% of production animals in developing countries are suffering from parasitosis. The pathogenic significance of GI parasitism includes poor body condition score (BCS), reduced growth with poor production of milk and meat, and, in severe conditions, death [9]. While parasitic infections are acting sub-clinically, they are causing enormous financial losses to marginal farmers [22]. An estimated annual cost of helminthiasis in dairy goats is 67–107 million euros in Europe [23]. Comparatively, GI parasitosis is more interested in reducing the productive performance of the goat than in causing death.

Parasitism is the ultimate outcome of the interaction of host factors, parasite factors, and environmental factors. There are several studies reflecting the critical role of host age, sex, body condition, housing facilities, medication, and grazing provisions [9,17,19,24–28]. Determination of the accurate role of these risk factors helps to develop a mitigation plan and restricts communication between the host and parasite, leaving livestock less susceptible. Although epidemiological studies have been conducted in other parts of Bangladesh, the prevalence and associated risk factors for GI parasitism in BBGs in Natore have yet to be investigated. Therefore, this study estimated the parasite diversity, infection rate, and factors related to GI parasitism in BBGs in different upazilas of the Natore district, Bangladesh.

## Materials and Methods

### Ethical consideration

Before the beginning of the experiment, verbal consent was taken from each of the animal owners in this study. Animal welfare issues were considered while sampling.

### Study area

**Figure 1.**
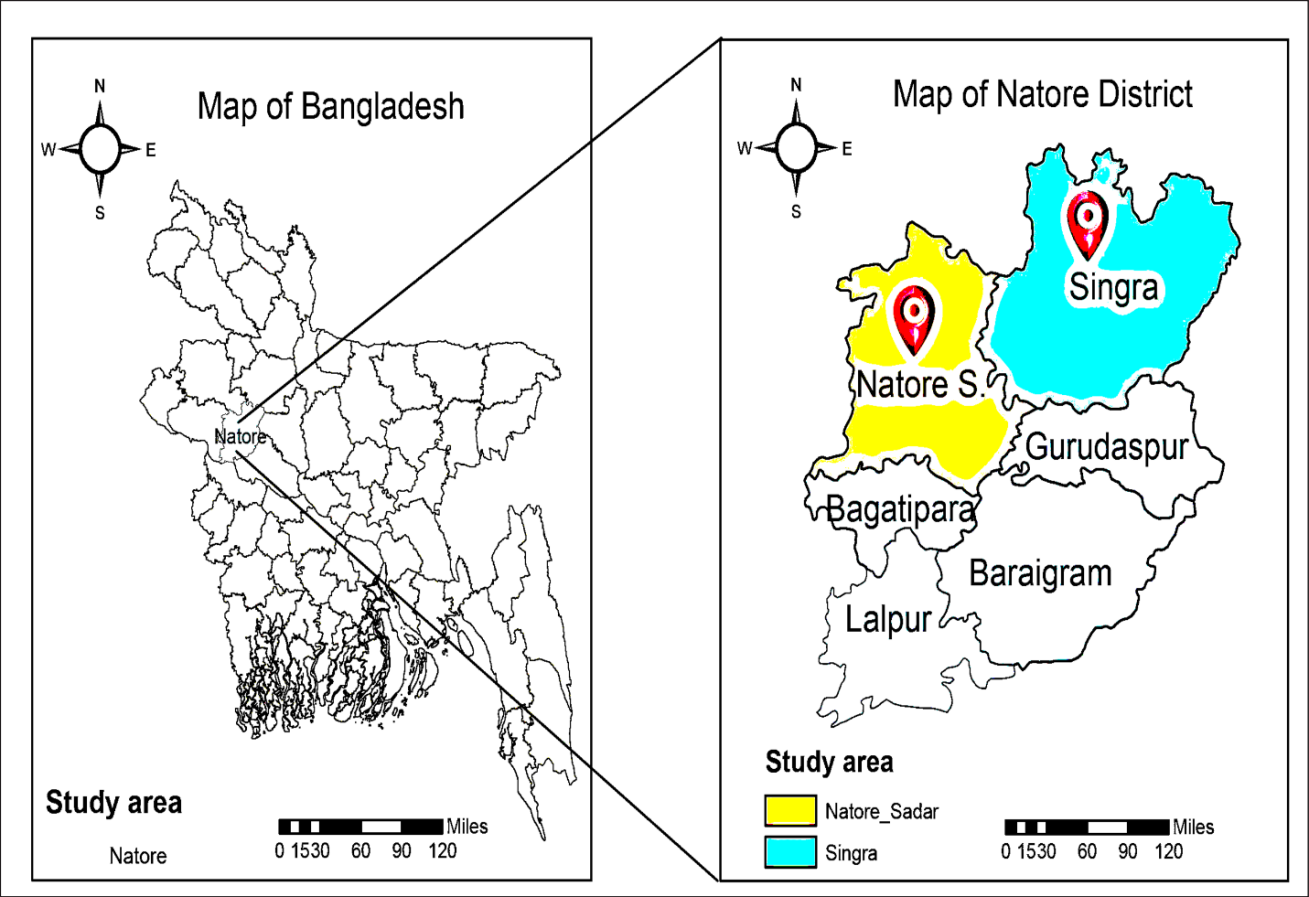
Map of the study area, Natore district and the sampling locations.

The selected study areas were Sadar and Singra upazilas under the Natore district, and the study was conducted from January 2017 to June 2017. As per Banglapedia, Natore, a district of the Rajshahi division, is 1,896.05 km^2^ (Fig. 1) [11]. On average, maximum 37.8°C and minimum 11.2°C temperatures have been reported yearly, along with annual rainfall of 1,862 mm [29]. In Natore, BBGs are most commonly reared without any proper management technique. Fecal samples were collected from different villages in the Dighapotia, Harishpur, Hatiandaha, and Kalam unions. Identification and other experimental works were performed in the laboratory of the Department of Parasitology, Bangladesh Agricultural University, Mymensingh.

### Sampling strategy

Fecal samples were collected using a simple random sampling technique. Initially, the sample size was 288, and we used the following formula, *n =* 1.962 (*P*_exp_ (1 − *P*_exp_))/*d*^2^, where *d* = desired precision, *n =* sample size, and p = expected prevalence [30]. We used a precision of 5% (*d* = 0.05), a confidence level of 95% (i.e., 1.96), and a 75% expected prevalence (p = 0.75) as per the available data from our study. However, finally, 260 goats were included in the study because 28 household owners refused to cooperate.

### Questionnaire survey

A survey was done with a questionnaire that asked about the host and how the goats were raised. After observing the animals and interviewing the owners, data were collected. The queries and observations were documented for statistical analysis purposes. The variables included age, sex, body condition (e.g., BCS), the housing system, and the use of an antihelmintic. Age was classified into two groups: 6 months to 1 year and >1 year, following the dentition chart and interviewing the farmers. Based on the BCS, BCS > 2 and BCS ≤ 2 were considered good and poor, respectively. After visiting the farmers’ homes, the rearing system was classified as backyard and semi-intensive; housing was divided into muddy and concrete/slatted. The status of anthelmintic treatment was recorded by interviewing the owners of the animals.

### Collection and processing of sample

Approximately 3–5 gm of feces were collected directly from the rectum or when freshly voided. Samples were stored in plastic containers with 10% formalin for preservation and refrigerated at 4°C until further use with proper labeling. For microscopic identification, nematode/cestode eggs and protozoan oocysts were recovered using the flotation method. Trematode eggs were obtained by applying a simple sedimentation technique as per the protocol described by Zajac and Conboy [31]. The same protocol was applied to determine fecal egg counts, where modified McMaster and modified Stoll’s Ova Counting techniques were performed to identify nematode eggs, protozoan oocysts, and trematode eggs, respectively [31]. Implementing the keys and description provided by Foreyt [32], helminth eggs and protozoan oocysts were identified. Each sample was examined three times to avoid miscalculations. 

### Statistical analyses

After the collection of the data, analyses were performed by the Statistical Package for Social Sciences using the *F* test. To identify whether the prevalence of parasites differs in both sexes or not, sex-related data were analyzed using a paired sample *t*-test [33]. The odd ratio was calculated using the formula given by Szumilas [34].

## Results

### Overall prevalence of GI parasites of BBGs in Natore

**Figure 2.**
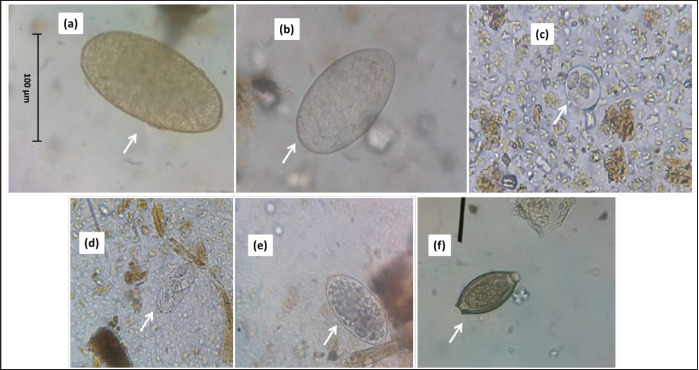
Eggs and oocyst of identified GI parasites in goats, (a) egg of *F. gigantica*, (b) egg of *Paramphistomum* spp., (c) sporulated oocyst of *Eimeria* sp., (d) egg of *Strongyloides* sp., (e) egg of *Haemonchus* sp., and (f) egg of *Trichuris* sp. (40× magnifications).

In this study, 65.4% (170/260) BBGs were found to be infected with single or multiple species of GI helminths (Table 1). Six different types of parasites were recovered (Fig. 2), where two species were trematodes, namely *Fasciola gigantica* (8.5%), *Paramphistomum* spp. (21.5%); three species of nematodes, namely *Haemonchus* spp. (20%), *Strongyloides* spp. (34.2%), *Trichuris* spp. (8.5%); and only one protozoan, namely, *Eimeria* spp. (9.2%). In this study, the range of Egg/gm (EPG) or Oocyst/gm (OPG) in the feces was 100–1,200. The highest EPG was counted in the case of *Strongyloides* spp. (900), followed by *Haemonchus* spp. (450), *F. gigantica* (300), *Paramphistomum* spp. (300), *Eimeria* spp. (350), and *Trichuris* spp. (200). *Strongyloides* spp. (66.2 ± 7.2) obtained the maximum mean EPG count, followed by *Paramphistomum* spp. (37.3 ± 4.9), *Haemonchus* spp. (25.4 ± 3.5), *Eimeria* spp. (12.3 ± 2.6), *F. gigantica* (10.8 ± 2.5), and *Trichuris* spp. (9.6 ± 2.1). 

**Table 1.** Overall prevalence of GI parasites of goats in Natore as detected by fecal sample examination (*n =* 260).

Name of GI parasites	No. of goats affected	Prevalence (%)	EPG	
			Range	Mean ± SE
*F. gigantica*	22	8.5	100–300	10.77 ± 2.46
*Paramphistomum *spp.	56	21.5	100–300	37.31 ± 4.97
*Strongyloides *spp.	89	34.2	100–900	66.15 ± 7.21
*Haemonchus* spp.	52	20	100–400	25.38 ± 3.51
*Trichuris *spp.	22	8.5	100–200	9.62 ± 2.06
*Eimeria* spp.	24	9.2	100–300	12.31 ± 2.61
Total	170^a^	65.4	100–1,200	161.15 ± 10.35

^a^ Total number of animals affected is less than the summation of individual infection because same animal was infected with more than one type of gastro-intestinal parasites.

### Host factors related to GI parasitic infections of goats in Natore 

In this study, age, sex, and body condition had no marked (p > 0.05) effect on the infection rate of a parasitic infection of BBGs (Table 2). The prevalence of GI parasitic infections was almost equal in young (65.6%) and adult goats (65.2%). But the severity of infections was higher in adult goats (175.6 ± 16.2) than in young goats (145.6 ± 12.5). The study revealed that the female (70.9%) goats were more vulnerable than the male (63.0%) goats. Female goats were 1.43 times more susceptible than female goats. The mean EPG and OPG were also higher in female goats (168.5 ± 13.5) than in male goats (144.3 ± 14.2). In this study, it was observed that poor-body-conditioned goats (74.5%) were less resistant to susceptibility to GI parasitic infections than good-body-conditioned goats (63.2%). Poorly body-conditioned goats were 1.70 times more susceptible than the good ones. Poorly body-conditioned goats (188.2 ± 19.5) had a larger mean EPG count range than that of well-body-conditioned goats (154.6 ± 11.9). 

### Management factors related to GI parasitic infections of BBGs in Natore 

The way goats are raised and where they live did not have a big effect on GI parasitic infections (*p* > 0.05; Table 3). Compared to semi-intensively reared goats (62.4%), free-range goats (72.2%) had more parasitic infections. The odds ratio was 1.55. The mean intensity of infection was also greater in freely roaming goats (196.2 ± 22.2) than in goats reared in a semi-intensive system (145.9 ± 11.1).

**Table 2.** Host factors related prevalence of GI parasites of goats in Natore as detected by fecal sample examination (*n =* 260).

Risk factors		No. of goats affected	Prevalence (%)	EPG		Odds ratio	*p-*value
				Range	Mean ± SE		
Age	6 months–1 year (*n =* 125)	82	65.6	100–700	145.6 ± 12.52	1.01	0.52^NS^
	>1 year (*n =* 135)	88	65.2	100–1,200	175.56 ± 16.16		
Sex	Male (*n =* 79)	56	70.9	100–400	144.30 ± 14.15	1.43	0.27^NS^
	Female (*n =* 181)	114	63.0	100–1,200	168.51 ± 13.51		
Body condition	Poor (*n =* 51)	38	74.5	100–400	188.24 ± 19.53	1.70	0.17^NS^
	Normal (*n =* 209)	132	63.2	100–1,200	154.55 ± 11.94		

NS = Not significant (*p* > 0.05), *n =* No. of goat examined.

**Table 3.** Management factors related prevalence of GI parasites of goats in Natore as detected by fecal sample examination (*n =* 260).

Risk factors		No. of goats affected	Prevalence (%)	EPG		Odds ratio	*p-*value
				Range	Mean ± SE		
Farming system	Free- range (*n =* 79)	57	72.2	100–1,200	196.20 ± 22.24	1.55	0.16^NS^
	Semi-intensive (*n =* 181)	113	62.4	100–800	145.86 ± 11.12		
Housing	Muddy (*n =* 225)	152	67.06	100–1,200	166.67 ± 11.16	1.96	0.09^NS^
	Concrete (*n =* 35)	18	51.4	100–700	125.71 ± 27.29		
Anthelmintic treatment	Non-treated (*n =* 197)	168	85.3	100–1,200	209.64 ± 11.52	176.68	<0.001^*^
	Treated (*n =* 63)	2	3.2	100–300	9.52 ± 6.68		

* Statistically significant (*p* < 0.05).

NS = Not significant (*p* > 0.05); *n =* No. of goat examined.

In this study, usually, two types of goat houses were noticed: one with a brick-built concrete floor and another with a muddy floor. Most GI parasitic infection was found in goats kept on muddy floors (67.1%) instead of concrete floors (51.4%). The odds ratio between a muddy and a concrete floor indicated that goats kept on the former were 1.96 times more susceptible to parasitosis than goats reared on the latter. The severity of infections was also higher in goats kept on muddy floors (166.7 ± 11.16) than in goats kept on concrete floors (125.7 ± 27.3).

In this study, deworming had an impressive influence on the availability of enteric infections in goats (*p* < 0.001). Anthelmintic-treated goats (3.2%) were almost free of GI parasitism compared to untreated goats (85.3). The mean EPG was also higher in non-dewormed goats (209.6 ± 11.5) than in dewormed goats (9.5 ± 6.7).

## Discussion

Goats are considered cows by farmers whose economic status does not permit them to raise a cow, and they also play a great role in daily nutrition at a cheaper cost [2]. BBGs have long been recognized for being highly prolific and disease-resistant [6]. Despite the genetic potential for resistance to many diseases, they are susceptible to many parasitic infections, including GI parasites, in the study area of Bangladesh, with a prevalence of 65.4%. Natore district is exclusively reputed for numerous rivers, *beels*, and small water bodies [11,29], which are enriched with different aquatic plants. These freshwater sources can serve as a potential habitat for vectors of helminth parasites (e.g., *F. gigantica*, *Paramphistomum* spp.). These plants are often handled as farm animal feed through grazing near water sources or manual processing. In addition, these areas are frequently flooded, leading to a greater chance for the dissemination of GI nematodes and host immunosuppression. The interaction of several factors could have contributed to the high percentage of parasitic infections in BBGs in the study area. The findings of this study indicate that *Strongyloides* were highly prevalent (34.2%), followed by *Paramphistomum* spp. (21.5%), *Haemonchus* spp. (20%), Eimeria spp. (9.2%), Trichuris spp. (8.5%), and *F. gigantica* (8.5%). This outcome supports the previous findings of Hassan et al. [18], Dhara et al. [24], Brahma et al. [35], and Dey et al. [9], who reported 63.41%, 62.34%, 73.34%, and 62.1% of goats infected with helminths, respectively. Higher rates of enteric infections were observed by Nath et al. [17], Rabbi et al. [19], Hassan et al. [25], and Wuthijaree et al. [36], who reported 94.67%, 76.5%, 89.33%, and 87.2%, respectively. Relatively lower infection rates were reported by Hossain et al. [12], Akther et al. [20], Das et al. [37], and Yasin et al. [13], who reported 9.7%, 28.65%, 56.3%, and 51.1%, respectively. Factors like variation in an experimental setting, sample size, and diagnostic techniques, besides ecological and host-pathogen factors, might have contributed to the deviation among the findings of different researchers. Moreover, the true prevalence could be higher than the rate revealed from the copro-microscopic examination of this study because of the high chances of missing larval stages or low-grade infections in such diagnostic procedures. 

Nematodes can have a direct life cycle and live in the environment for a long time in different forms and stages, such as when they are active. Some forms are very hardy and can stay alive for up to 10 years even if the environment or other things try to kill them [38]. These biological features of nematodes, especially geohelminths, help them live longer, infect more hosts, and spread to more hosts. This makes them more common, as our study for *Strongyloides* and *Haemonchus* shows. Besides, Dey et al. [9] and Dhara et al. [24] reported comparatively lower prevalence of *Trichuris* in Bangladesh and India, respectively. Trematodes are mainly vector snail-dependent to complete their life cycle. Both *F. gigantica* and *Paramphistomum* spp. require aquatic snails as intermediate hosts to complete their life cycles. The grazing of goats in areas with water bodies or low-lying grassland submerged during the rainy season or flood, as well as the presence of specific vector snails, supports the animals’ infection with metacercariae of trematodes in the grass blades. Rabbi et al. [19] and Dhara et al. [24] recorded 14.8%. They reported 6.29% *Fasciola* infectivity in Bangladesh and India, respectively, similar to the prevalence of *F. gigantica* infection in the present study. In their study, Rabbi et al. [19] discovered that 28.5% *Paramphistomum* spp. infection. Ruminants are highly affected by the protozoan parasite *Eimeria* spp., and this infection rate could be as high as 90% [39]. High stocking density because of the narrow animal shed and shared accommodation with other domestic animals, breeding intensification, and other physiological stress may promote coccidiosis in goats [40]. Species diversity and differences among various biotic and abiotic variables in GI parasitosis might be regulated by environmental, host-related, and parasite-related issues. Variations in topography, environmental conditions, age, sex, breed, body condition, stress, availability of snail intermediate hosts, greenery, grazing technique, rearing and management measures, cohabitation with other susceptible livestock species, deworming, genetic resistance, etc. can potentially control the frequency and intensity of GI parasitism [9,41].

Several recent studies have revealed that host age, sex, and body condition all have a significant impact on the status and intensity of GI parasitism [9,12,27,28]. Contrary to this, our study found no significant effect of age, gender, or body condition on endo-parasitic infection in BBGs. Among the age groups, young goats were relatively more susceptible than adult goats, in accordance with Zvinorova et al. [28] but contrary to Singh et al. [27]. In the case of mixed infections, the reasons behind the frequent occurrence of parasitic infections in young and adult animals are still tough to define. Because of the worn-out immunity of adults and the naive immunity of the young, this might lead to low resistance or greater host susceptibility in addition to the higher scope of cross-transmission. BBGs of the male sex were less affected in our study than those of the female sex, which is consistent with the findings of Singh et al. [27] and Hossain et al. [12] but not with the findings of Zvinorova et al. [28]. Considering host nutrition and, thereby, body condition, poorly body-conditioned hosts were 1.70 times more susceptible to GI parasitism than normally body-conditioned ones. Malnutrition in animals increases their susceptibility, whereas balanced nutrition might contribute to the development of resilience in the host through immunoregulation [41]. This study reports no significant effect of rearing systems (free-range and semi-intensive) and housing types (muddy floor and concrete floor) on parasitic infections in goats. But Rabbi et al. [19] reported an 86.1% prevalence in the semi-intensive system compared to 57.5% in the intensive system. In this study, animals kept on muddy floors (67.06%) were more affected than those kept on concrete floors (51.4%). The calculated odds ratio was 1.96, indicating that a muddy floor was 1.96 times more dangerous as a habitat than a concrete floor. The prevalence of helminth infection may be due to overcrowding, poor management, and hygiene [42]. Overcrowding and poor hygienic practices greatly encourage the spread of these parasites, as animals become carriers of parasites and contaminate the floor with eggs and oocysts of parasites. Cleaning a concrete floor is more frequent than cleaning a muddy floor. So, the possibility of parasitic infection is high.

Although anthelmintic resistance is rapidly growing globally, including in Bangladesh [43,44], routine anthelmintic deworming has long been used as endoparasite control. This study found that deworming could significantly reduce parasites in caprine. Goats that were not treated with anthelmintic (85.3%) were found to be more highly infected than goats that were treated with anthelmintic (3.2%). These results are related to the previous findings of Salgado and Santos [45], who reported that systematic and appropriate use of antiparasitic drugs in small ruminants effectively controls parasitic diseases. The findings partially agree with Belina et al. [46], who showed that risky practices, including the professionally unsupervised prescription and use of anthelmintic drugs and improper dosing of anthelmintic drugs, might contribute to the existence of circulating parasite species in the hosts. Such malpractices were also common in the study area. The prevalence of GI parasites can be reduced by emphasizing the farmer’s perception of the impact of parasitic infection and anthelmintic utilization. 

## Conclusion

Goat rearing is an integral part of our livestock farming and entrepreneurship in rural and peri-urban life in Bangladesh, which is also rapidly emerging in these particular research areas. In Natore, BBGs were frequently and severely affected by GI helminths. This is a serious concern because the infection load in the environment is high, leading to greater susceptibility in the study area. On top of that, a higher prevalence of nematode infection was evident than that of trematodes and protozoa. The study revealed the vital role of anthelmintics in controlling parasitism, even with the emerging trend of anthelmintic resistance worldwide. Now it is time to optimize our strategy and decide whether to do routine deworming or make strategic changes. The findings of this study had limitations because we used copro-microscopy as the only method for parasite detection. Consequently, in many cases, species-specific identification was difficult. However, extensive investigation of parasitism using a larger population and more accurate diagnostic tools is suggested. In addition, practical manuals on animal husbandry and anthelmintic use must be developed to minimize parasitism and anthelmintic resistance worldwide.

## List of abbreviations

BBGs, Black Bengal Goats; GI, Gastrointestinal; EPG, Egg per gram of feces.
